# Treatment of Localized Gingival Recession with an Enamel Matrix Protein-Coated Xenogeneic Dermal Matrix: A Randomized Controlled Trial

**DOI:** 10.3390/ma17163985

**Published:** 2024-08-10

**Authors:** Marcus Rieder, Gernot Wimmer, Alwin Sokolowski, Armin Sokolowski, Michael Payer, Behrouz Arefnia

**Affiliations:** 1 Division of Oral and Maxillofacial Surgery, Department of Dental Medicine and Oral Health, Medical University of Graz, 8036 Graz, Austria; marcus.rieder@medunigraz.at; 2 Division of Restorative Dentistry, Periodontology and Prosthodontics, University Clinic of Dental Medicine and Oral Health, Medical University of Graz, 8036 Graz, Austria; gernot.wimmer@inode.at (G.W.); alwin.sokolowski@medunigraz.at (A.S.); armin.sokolowski@medunigraz.at (A.S.); 3 Division of Oral Surgery and Orthodontics, University Clinic of Dental Medicine and Oral Health, Medical University of Graz, 8036 Graz, Austria; mi.payer@medunigraz.at

**Keywords:** coronally advanced flap, collagen, enamel matrix protein, dental enamel protein, gingival recession, periodontal plastic surgery

## Abstract

This study aims to evaluate the influence of the additional use of enamel matrix derivate (EMD) in the treatment of gingival recession defects using a coronally advanced flap (CAF) and a xenogeneic dermal matrix (XDM) by means of digital and clinical assessment methods. In this prospective randomized controlled study, recession height and area, width and thickness of keratinized gingiva, pocket probing depth, and clinical attachment levels were measured at the baseline and followed up for one year. Fifteen patients (*n* = 15) with 24 gingival recession defects were treated between 2019 and 2021. On average, the digitally assessed root coverage of the control group (CAF + XDM) was not significantly different compared to the test group (CAF + XDM + EMD), with 69 ± 28% and 36 ± 32%, respectively (*p* = 0.094). One year postoperatively, there were no differences found regarding keratinized tissue width (KTW) between the control group and test group (*p* = 0.690). However, the control group showed superior results in the thickness of keratinized gingiva (*p* = 0.044). The present study showed that there were no statistically significant differences in the root coverage results in the CAF + XDM + EMD group compared to the CAF + XDM group. The adjunctive use of EMD to a CAF and XDM in the treatment of gingival recession defects does not appear to have any clinical benefit.

## 1. Introduction

While the etiology of gingival recession is multifactorial, untreated recession defects often progress despite maintaining proper oral hygiene [1,2]. Between 10% and 30% of the population is affected by dentin hypersensitivity, mostly due to exposure of dentin caused by gingival recession [3]. If there are no aesthetic complaints, a nonsurgical treatment plan should be attempted first before considering a surgical option [4]. There are several toothpastes that alleviate hypersensitivity by either inhibiting nerve conduction or occluding dentinal tubules. Another non-invasive option in the treatment of tooth hypersensitivity is local administration of dentin desensitizers [5]. In cases where patients experience impaired aesthetics or persistent dentin hypersensitivity, surgery becomes the treatment of choice [6]. The decision to select a particular surgical technique is dependent on several factors, some of which are related to the defect morphology (recession classification, clinical attachment level, width of keratinized gingiva, gingival phenotype, presence of frenula or muscle traction, and depth of the vestibule), while others are related to the patient (patient’s expectation, compliance, and systemic and local conditions, such as smoking, tooth brushing, plaque control, or pregnancy) [7,8]. Regardless of the surgical technique, adjuvant postoperative home management, such as mouth rinses, may further improve the outcome [9,10,11].

A meta-analysis, which combined the results of 48 randomized trials from different periodontal plastic surgery techniques, concluded that subepithelial connective tissue grafts (SCTGs) and coronally advanced flap (CAF), alone or in combination with other biomaterials and guided tissue regeneration, may be used as root coverage procedures. However, it was reported that more randomized controlled trials were necessary to associate possible factors with the prognosis of each procedure. Nevertheless, the study emphasized that the utilization of SCTGs demonstrated improved results regarding the increase in root coverage and keratinized tissue width (KTW) compared to the other treatment modalities [12]. However, the harvesting procedure is associated with pain and complications (e.g., haemorrhage, necrosis) at the donor site [13]. Although a SCTG is considered the gold standard in the treatment of single and multiple gingival recession defects, xenogeneic dermal matrices (XDMs) have been proposed as a substitute to reduce the patients’ donor site morbidity [12,14,15,16,17]. The use of EMD in periodontal plastic surgery is based on its ability to regenerate lost periodontal tissue, as demonstrated in several studies of both humans and animals [18,19,20,21,22]. Furthermore, a histological study on minipigs revealed that the use of EMD can improve periodontal regeneration regardless of its combination with a collagen matrix [16].

Hence, the present trial aims to compare periodontal plastic surgery using a CAF + XDM (Mucoderm^®^, Botiss, Zossen, Germany) versus a CAF + XDM +EMD (Emdogain^®^, Straumann, Basel, Switzerland) in the treatment of gingival recession. The null hypothesis of this study is that the additional use of EMD for periodontal plastic recession surgery with CAF + XDM is not superior in terms of clinical outcomes.

## 2. Materials and Methods

### 2.1. Trial Design

This prospective randomized controlled study was conducted at the tertiary clinical centre of the Medical University of Graz, Austria and registered in the European Union Clinical Trials Register (NCT05799859). Two surgical approaches were compared to treat gingival recession defects in the upper and lower jaw: group control (CAF + XDM) and group test (CAF + XDM + EMD). The clinical trial was carried out following all local legal requirements and the Declaration of Helsinki (1975) as well as the approval of the Ethics Committee of the University (IRB00002556, No.: 28-123 ex 15/16). Prior to the start of the study, written informed consent was obtained from all subjects before treatment. All surgical procedures and complete follow-up visits were conducted at the Medical University Graz. No changes to methods were made after trial commencement.

### 2.2. Participants

The study included a total of 15 patients (group control: *n* = 7, group test: *n* = 8) from the University Clinic of Dentistry and Oral Health, Medical University Graz.

### 2.3. Eligibility Criteria

Individuals: (1) over 18 years old; (2) with signed informed consent; (3) presenting with at least one gingival recession defect (categorised as Miller class I or II/Cairo class recession type (RT)1) [23,24,25]; (4) with the presence of a visible cemento-enamel junction; (5) with no prior experience of root coverage procedures; and (6) able to achieve good oral hygiene (full mouth plaque score (FMPS) < 20%, full mouth bleeding score (FMBS) < 20%) were invited to participate.

### 2.4. Exclusion Criteria

Exclusion criteria comprised: (1) noncarious cervical lesions; (2) general contraindications to dental surgery under local anaesthesia; (3) ongoing or previous chemotherapy, radiotherapy, or bisphosphonate therapy; (4) smokers; (5) pregnancy and nursing mothers; (6) disorders or treatments that impair wound healing; (7) long-term treatment with high-dose steroids or anticoagulants; (8) bone metabolism disorders; (9) infections or vascular disorders; (10) known hypersensitivity to porcine collagen; and (11) extruded or malpositioned teeth.

### 2.5. Preparation Period

Prior to surgery, all patients enrolled in the study received a full periodontal examination (i.e., probing pocket depth (PPD), FMBS, FMPS, KTW, and recession height (RECH)), followed by periodontal therapy consisting of oral hygiene instructions and dental prophylaxis. If necessary, deep scaling and root planing were performed. Four weeks after initial therapy, all patients underwent a second periodontal examination.

### 2.6. Clinical and Digital Assessment

The clinical assessment was conducted prior to surgery, as well as one, three, six, and twelve months postoperatively. PPD (i.e., distance from gingival margin to bottom of gingival sulcus), RECH (i.e., distance from cemento-enamel junction to gingival margin) and KTW (i.e., distance between gingival margin and muco-gingival junction) were measured using a calibrated, constant pressure electronic periodontal probe (Florida Probe^®^, Florida Probe Corp., Gainesville, FL, USA). PPD and RECH were used to determine the clinical attachment level (CAL). All clinical measurements were performed by one calibrated and masked periodontist (BA) not involved in the surgery. The process of calibration was executed by dual measurements of 40 gingival recession defects on plaster models. Regarding intra-examiner reproducibility, BA reached an intraclass correlation coefficient of 0.88.

Intraoral scans were taken in all patients prior to surgery and six months postoperatively. These scans were used digitally to evaluate the RECH, recession area, as well as the keratinized tissue thickness (KTT). 3D-Slicer^®^ (Version 5.4.0, Slicer Community, Bethesda, MD, USA [26]) and OraCheck^®^ software (Version 5, Dentsply Sirona, Charlotte, NC, USA) were used to analyse the intraoral scans.

### 2.7. Randomization and Treatment

Following patient selection, group allocation (test or control) was performed using the Randomizer for clinical trials^®^ software (Version 2.1.0, Institute for Medical Informatics, Statistics and Documentation, Medical University of Graz, Graz, Austria) by an individual not involved in patient recruitment, treatment, or evaluation. Following this step, sealed envelopes were created which contained the assigned codes for each patient’s randomized treatment. All surgical procedures were performed by an experienced periodontal surgeon (GW) at the University Clinic of Dentistry and Oral Health, Medical University Graz. A #15 blade was used to prepare the mucosal split-full-split flap. After de-epithelialization of the papillae, root preparation was performed with hand instruments, rotary instruments, or ultrasonic instruments (HuFriedyGroup, Frankfurt, Germany). A fine-grained diamond bur (Perio-Set^®^, Intensiv SA, Grancia, Switzerland) was used to remove convexities, indentations, and sharp edges on the root surface. Thereafter, the treatment was revealed to the surgeon. After measuring the gingival recession defect by means of a periodontal probe, a rehydrated XDM of an appropriate size was cut. Fixation of the matrix was done with 6.0 absorbable single button suture (PTFE Omnia, HuFriedyGroup, Frankfurt, Germany). The CAF was fixed at least 1 mm coronal to the cemento-enamel junction with 6.0 monofilament non-absorbable single button sutures. All previously described steps were performed in both groups. However, the test group received a synthetically produced 24% EDTA liquid (PrefGel^®^, Straumann, Basel, Switzerland), which was applied for 2 min after root planing and then completely rinsed off with a physiological saline solution. Thereafter, EMD was applied to both the root surface and to the XDM.

Patients were instructed to abstain from brushing the treated area and to rinse their mouths twice daily with a 0.12% chlorhexidine solution for 2 weeks. Anti-inflammatory therapy was administered for a minimum of two days (e.g., ibuprofen 400 mg) and was extended if required. Suture removal was completed 10 days after the procedure.

### 2.8. Statistical Analysis and Data Management

All data were deidentified before usage and stored in a protected Microsoft Excel^TM^ database (Version 16.70, Microsoft Excel for Mac, Microsoft, Redmont, WA, USA). Descriptive and analytical statistics were employed to analyse the parameters of this study. Analytical statistics included the *t*-test, mixed model test, and the general linear model with repeated measures, which were used when appropriate after normality distribution was checked by the Shapiro–Wilk test. For all calculations, a *p*-value < 0.05 was considered statistically significant. All statistical analyses were performed using the SPSS statistical software package (Version 24.0, SPSS Statistics for Windows, Microsoft, Armonk, NY, USA).

The recorded data were collected in a case report form. The collection, transfer, storage, and analysis of personal data within this study were carried out in accordance with legal regulations.

### 2.9. Sample Size Calculation

Sample size calculation was performed considering RECH as the primary outcome. The study was powered to detect a minimum clinically significant difference in root coverage of 1.0 mm with an assumption of α = 0.05 and power = 95%, based on data from a previous study [27,28]. Eleven recessions per treatment arm were needed in the present trial. Considering dropouts, a total sample of 24 recessions were enrolled to achieve the power of the study. No subjects were lost up to the follow-up at 6 months.

## 3. Results

**Figure 1:**
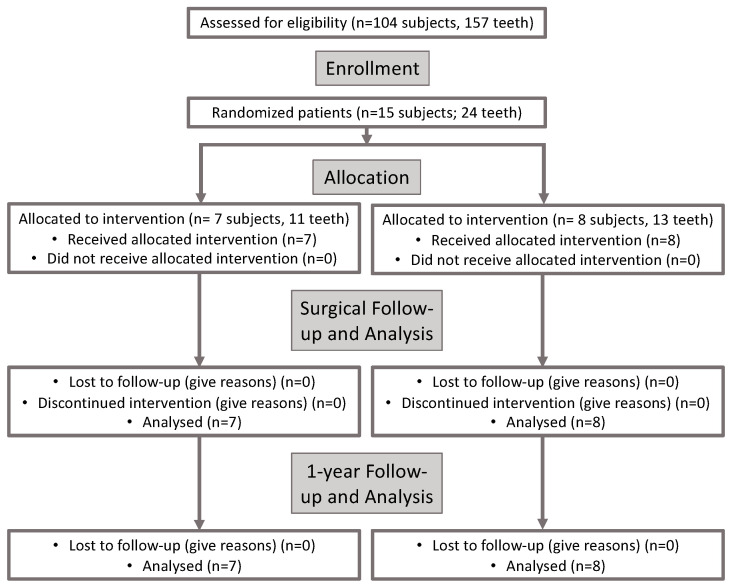
CONSORT diagram shows the overall steps and design of this study.

Out of 105 people assessed, a total of 15 patients (*n* = 15) were enrolled in the main trial, with seven patients (*n* = 7; 47%) randomized for the control group and eight patients (*n* = 8; 53%) for the test group (Figure 1). Of these 15 patients, 24 teeth (*n* = 24) were treated with periodontal plastic surgery (CAF + XDM: *n* = 11; CAF + XDM + EMD: *n* = 13). The recruitment period was from October 2019 to September 2021. Participation in the study lasted 13 months per subject.

Patient characteristics did not differ between the control and the test group, with a similar mean age of 30.4 ± 4.6 and 31.2 ± 8.2 years (*p* = 0.823) and a similar mean body mass index (BMI) (control group: 20.2 ± 2.2; test group: 20.7 ± 1.6; *p* = 0.583) and sex (*p* = 0.182). All patients had a full-mouth bleeding and plaque score of <20% at the time of the surgery. One gingival recession worsened after the periodontal plastic surgery was conducted. No patients were lost to follow-up during the trial. The demographic case characteristics are presented in Table 1.

### 3.1. Clinical Assessment

Mean PPD in the control group was 1.00 ± 0.00 mm, and it was 1.60 ± 0.77 mm in the test group before treatment. Following the periodontal plastic surgery procedure, the PPD did not change in the control group and decreased to 1 mm in the test group. The mean CAL was initially equivalent with 5.46 ± 1.51 mm and 5.36 ± 1.63 mm in the control and test group, respectively. Concerning the distance of the two courses independent of the gradient, the general linear model with repeated measures showed no statistically significant difference (*p* = 0.083). Nevertheless, both groups showed a significant decrease of CAL over time (*p* < 0.001) and over gradient (*p* < 0.002). The mean clinically measured preoperative RECH was similar between the control and test group, with 3.91 ± 1.22 and 3.77 ± 1.01, respectively. The general linear model with repeated measures showed a statistically significant change of RECH over time and gradient for both groups (*p* < 0.001). With reference to the distance of the two courses independent of the gradient, there was no statistically significant difference identified (*p* = 0.071).

**Figure 2:**
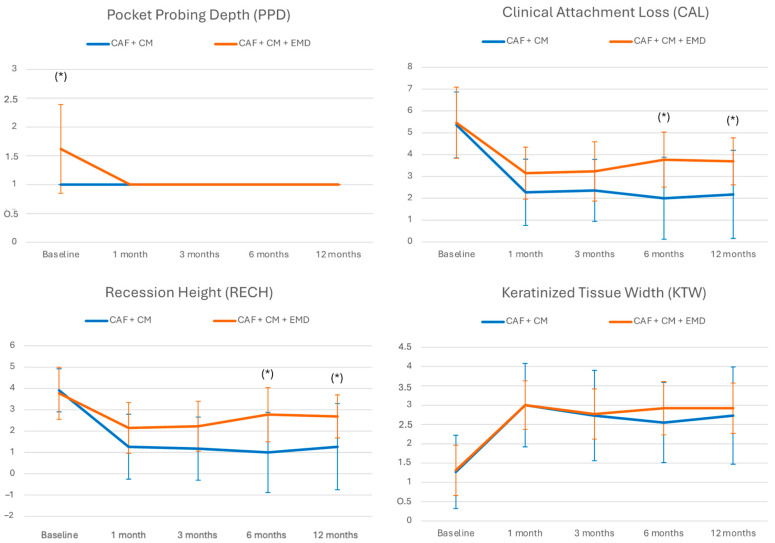
Clinical assessment of mean pocket probing depth, recession height, clinical attachment level, and width of keratinized gingiva over time measured in mm. * = intergroup significance.

The mean KTW apical to the gingival recession defect was 1.27 ± 0.65 mm in the control group and 1.31 ± 0.95 mm in the test group. After periodontal plastic surgery, the KTW increased in both groups significantly over time (*p* < 0.001). There was no significant alteration regarding the gradient over time (*p* = 0.074) or difference between the groups (*p* = 0.690). Figure 2 and Table 2 illustrate the clinical assessments of PPD, CAL, RECH, and KTW over the follow up period.

### 3.2. Digital Assessment

**Figure 3:**
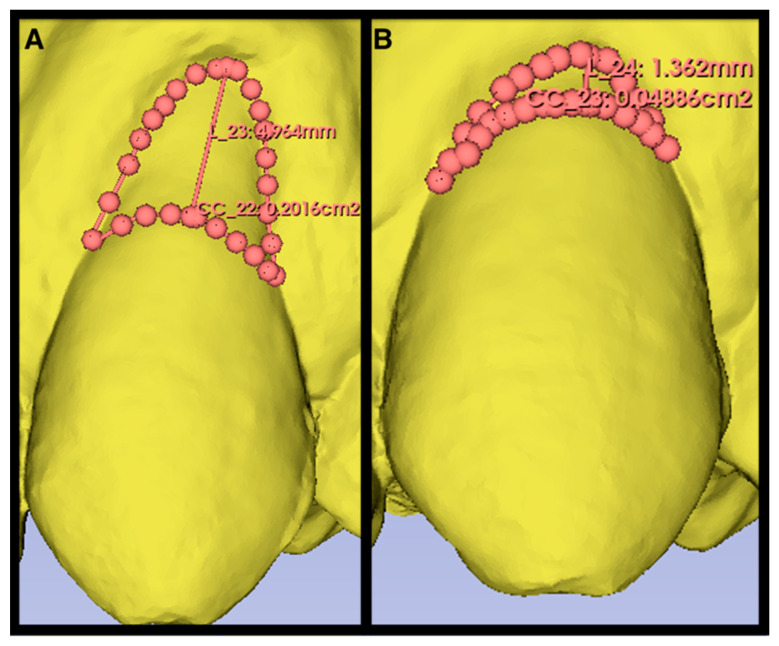
Digitally-obtained surface of the exposed root. (**A**) Preoperative scan of a 40-year-old patient with a recession height of 4.96 mm and a recession area of 20.16 mm^2^. (**B**) Six month postoperative scan with a recession height of 1.36 mm and a recession area of 4.88 mm^2^.

**Figure 4:**
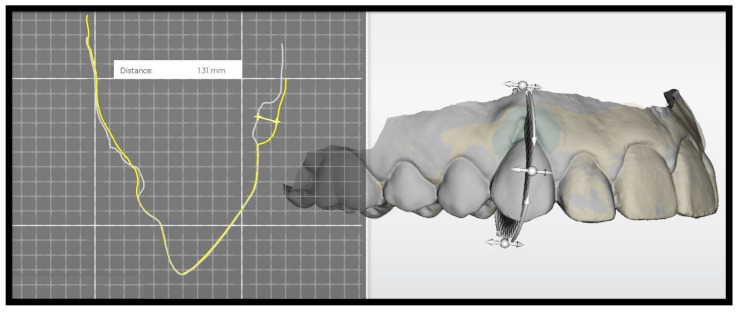
Digitally-obtained change in soft tissue thickness using OraCheck^®^ software by Dentsply Sirona. The grey (preoperative) and yellow (postoperative) lines indicate the sagittal plane of the treated tooth recorded by the intraoral scan.

Digitally measured recession defects assessed from the intraoral scans at baseline showed a mean RECH of 3.66 ± 1.35 mm in the control group and 3.60 ± 1.09 mm in the test group. Six months after surgery, the RECH were reassessed and showed an improvement to 1.18 ± 1.07 mm and 2.47 ± 1.47 mm, respectively. This corresponds to a root coverage of 69 ± 28% in the control group and 36 ± 32% in the test group. There is no statistically significant difference between the groups regarding root coverage; both measured as the difference in RECH between baseline and six months postoperatively (*p* = 0.054). Preoperatively, the mean area of recession (i.e., exposed root surface) was 11.45 ± 6.01 mm^2^ and 13.95 ± 5.23 mm^2^ in the control and test groups, respectively. Six months postoperatively, the mean exposed root surface area decreased to 3.02 ± 2.57 mm^2^ in the CAF + XDM group and to 10.59 ± 7.01 mm^2^ in the CAF + XDM + EMD group (*p* = 0.065). The postoperative KTT covering the root surface was measured in the mid sagittal plane of the treated tooth. In the control group, the KTT measured 1.05 ± 0.44 mm and ranged from 0.50 to 2.18 mm. The soft tissue thickness in the test group ranged from 0 to 1.22 mm with a mean value of 0.56 ± 0.41 mm. The mixed model test demonstrated a statistically significant difference between the CAF + XDM and the CAF + XDM + EMD groups with a *p*-value of 0.044 (Table 3). Figure 3 and Figure 4 show the digital workflow using 3D-Slicer^®^ and OraCheck^®^ software.

## 4. Discussion

In periodontal plastic surgery, there are several options to cover exposed root surfaces, using a variety of proven surgical methods and materials. The principal goal of all alternatives is to achieve predictable, long-term, and complete root coverage in conjunction with healthy gingival conditions and low patient morbidity. To reduce morbidity caused by SCTG harvesting and to allow simultaneous treatment of multiple recession defects, the use of XDM is a standard treatment option which offers several advantages for both the patient and surgeon. Various studies have already described the benefits of biomaterials in the treatment of gingival recession compared to CAF alone [12,27,29,30,31]. The biological intent of a collagen matrix (CM) is to act as a barrier against the proliferation of epithelial cells into the defect [22]. EMD is successfully used in a wide range of periodontal plastic surgery procedures, including root coverage, periodontal regeneration, and implant dentistry because of its ability to regenerate lost periodontal tissue, as demonstrated in several studies, in both humans and animals [21,31,32,33,34,35,36,37]. Additional application of EMD should improve the environment of periodontal-associated cells to promote regeneration and thereby better root coverage [35]. Histological evaluation showed formation of new bone, cementum, and periodontal ligament [18,19,20,21]. A histomorphometric study in minipigs revealed that the use of EMD can improve periodontal regeneration regardless of its combination with a collagen matrix [22]. This randomized controlled study included RT1 defects which were treated by a CAF technique using an XDM alone or in combination with an EMD.

Both tested approaches demonstrated a statistically significant improvement regarding the root coverage in RT1. Evaluating the RECH outcomes clinically with a periodontal probe showed stable results in both groups over the complete follow-up period (one year), and there was no detectable statistically significant difference between the two groups. Similar results were obtained when the digitally-obtained RECH and area of recession were compared. However, measuring the actual change of the exposed root surface may be more appropriate rather than measuring recession height.

The obtained root coverage results of this study are inferior when compared to the results of Sangiorgio et al. The varying inclusion criteria of the studies could be an explanation for these circumstances. In the present study, tooth type (incisor, canine, molar) and location (maxilla or mandible) did not play a role; however, in the study by Sangiorgio et al., only maxillary canines or premolars were treated. When comparing the two treatment groups in this study, three molars were treated in the test group and no molars in the control group. The more surgically demanding procedure for posterior teeth may have led to a worsening of the postoperative outcome, which would explain inferior results in the test group. In the test group, 9 out of 13 teeth were in the mandible, while in the control group, 9 out of 11 teeth were in the maxilla. The shallow vestibulum depth, the higher incidence of aberrant frenula with consecutive pull of the soft tissues, the inferior vascularisation compared to the maxilla, as well as the generally smaller surgical field (e.g., lower incisors) in the mandible may have led to a deterioration of root coverage. The difference between the two investigated groups demonstrates the importance of proper patient selection in periodontal plastic surgery.

In the present study, the KTT was determined digitally, which is a novel reliable assessment method without having to penetrate the gingiva [38,39]. The digitally-obtained results showed statistically significant superior results in the control group compared to the test group. A recent investigation by Chambrone and Tatakis described the positive effect of KTT on the long-term prevention of gingival recession [40]. There are several studies available which compare EMD to a combination of EMD and subcutaneous tissue grafts (SCTGs). One year postoperatively, Rasperini et al. reported a root coverage of 90% in the SCTG + EMD group and 80% in the STCG group [41]. In comparison, Roman et al. observed 82.3% for SCTG + EMD and 89.8% for SCTG alone [42]. In both studies, the concurrent use of EMD and SCTG did not show a significant additional benefit. While the use of EMD provides significant improvement over CAF alone, multiple combinations of biomaterials may offer similar or fewer benefits compared to periodontal plastic procedures using only one biomaterial [43]. However, a histologic examination of human buccal recession defects demonstrated that sites treated with EMD showed periodontal regeneration. In SCTG treated sites, this type of regeneration could not be found, despite similar clinical outcomes [19].

The present study demonstrates additional gain in KTW in both groups one year postoperatively (CAF + XDM: 1.61 mm, and CAF + XDM + EMD: 1.46 mm). This is in accordance with the results of Cardaropoli et al. and McGuire and Scheyer, which reported a KTW gain after periodontal plastic surgery using a CAF + CM of 1.23 and 1.34 mm, respectively [44,45]. Comparing CAF + CM with CAF, Jepsen et al. reported a superior outcome of the CAF + CM group, with 0.37 mm more KTW gain compared to the CAF group [29]. Evaluating the impact of EMD regarding the KTW, Cardaro et al. observed no impact using EMD (CAF: 0.31 mm, and CAF + EMD: 0.28 mm), whereas Del Pizzo et al. demonstrated a KTW gain over time (CAF: 0.47 mm, and CAF + EMD: 1.00 mm) [31,34]. Despite these findings, several authors have observed no statistically significant difference when comparing various treatment alternatives [27,28,46,47]. Furthermore, the type of xenogeneic matrix (CM versus XDM) does not seem to play a role regarding the outcome [48].

Although specific inclusion and exclusion criteria were applied, some limitations must be considered when interpreting the study’s results. The conducted prospective randomized controlled trial is one of the first investigations evaluating the periodontal plastic surgery technique using a CAF with an XDM and an EMD over a 12-month period [27,49]. In this study, the kind of teeth (i.e., incisor, canine, premolar, molar) were not an inclusion criterion. Therefore, the effects that result from the more surgically demanding procedure for posterior and mandible teeth were not evaluated in this study. This bias must be presented as it demonstrates the clinical relevance of patient and site selection for periodontal plastic surgery procedures. It additionally grants reasonable outcome predictions for both the patient and surgeon. Although the clinical evaluation was performed over a 12-month period, an evaluation utilizing the digital impression files after a period of healing of 12 months would have provided further insight. Future research projects could conduct a similar study on a split-mouth model with a larger sample size to explore this topic in depth. The null hypothesis of this study is accepted, stating that the additional use of EMD for periodontal plastic recession surgery with CAF + XDM is not superior in terms of clinical outcomes within the limitations of the study.

## 5. Conclusions

The present study indicated no statistically significant difference regarding root coverage results of the CAF + XDM + EMD group compared to the CAF + XDM group. Both surgical techniques showed gain regarding the KTW. Additionally, reliable evaluation methods of periodontal plastic surgery procedures were confirmed without the need to penetrate the gingiva, take impressions, or use a digital calliper tool.

## Figures and Tables

**Table 1:** Detailed description of patient characteristics. SD = standard deviation, BMI = body mass index.

	Control Group	Test Group	*p*-Value
Age			*p* = 0.823
min	26.0	19.9	
max	38.3	40.7	
mean	30.4	31.2	
SD	4.6	8.2	
Sex			*p* = 0.182
male	1 (14.3%)	3 (37.5%)	
female	6 (85.7%)	5 (62.5%)	
total	7	8	
BMI			*p* = 0.583
min	17.4	18.9	
max	24.0	23.1	
mean	20.2	20.7	
SD	2.2	1.6	
Tooth Distribution			
Upper Arch	9	4	
Canines	9	2	
Molars	0	2	
Lower Arch	2	9	
Incisors	2	2	
Canines	0	6	
Molars	0	1	

**Table 2:** Clinical assessment of the evaluated variables at baseline and 6 months postoperatively. SD = standard deviation, CAF = coronally advanced flap, CM = collagen matrix, EMD = enamel matrix protein, PPD = pocket probing depth, RECH = recession height, CAL = clinical attachment level, KTW = keratinized tissue width, mo = months, GLM = general linear model with repeated measurements.

		CAF+ CM		CAF + CM+ EMD		GLM			
		Mean	SD	Mean	SD	Intragroup Difference CAF+ CM	Intragroup Difference CAF + CM+ EMD	Intergroup Difference in Gradient	Intergroup Difference in Distance of Courses
PPD	baseline	1	0	1.62	0.77				
	6 months	1	0	1	0				
	12 months	1	0	1	0				
	6 mo—baseline	0	0	−0.62	0.77				
	12 mo—baseline	0	0	−0.62	0.77				
RECH	baseline	3.91	1.22	3.77	1.01				
	6 months	1.00	1.27	2.77	1.88				
	12 months	1.27	1.01	2.69	2.02	*p* < 0.001	*p* < 0.001	*p* < 0.001	*p* = 0.071
	6 mo—baseline	−2.91	1.30	−1.00	1.35				
	12 mo—baseline	−2.64	0.67	−1.08	1.66				
CAL	baseline	5.36	1.63	5.46	1.51				
	6 months	2.00	1.27	3.77	1.88				
	12 months	2.18	1.08	3.69	2.02	*p* < 0.001	*p* < 0.001	*p* = 0.002	*p* = 0.083
	6 mo—baseline	−3.36	1.63	−1.69	1.03				
	12 mo—baseline	−3.18	0.98	−1.77	1.36				
KTW	baseline	1.27	0.65	1.31	0.95				
	6 months	2.55	0.69	2.92	1.04				
	12 months	2.73	0.65	2.92	1.26	*p* < 0.001	*p* < 0.001	*p* = 0.074	*p* = 0.690
	6 mo—baseline	1.27	0.9	1.62	0.65				
	12 mo—baseline	1.45	0.69	1.62	0.77				

**Table 3:** Digital assessment of the recession (at baseline and 6 months postoperatively) and keratinized tissue thickness (6 months postoperatively). SD = standard deviation, mo = months.

	CAF + CM		CAF + CM + EMD		*p*-Values Intragroup		*p*-Values Intergroup
	Mean	SD	Mean	SD	CAF + CM	CAF+ CM+ EMD	
Recession Area (mm^2^) Baseline	11.45	6.01	13.95	5.23			*p* = 0.520
Recession Area (mm^2^) 6 mo Postoperatively	3.02	2.57	10.59	7.01			
6 mo—Baseline	−8.43	5.53	−3.36	3.16	*p* < 0.001	*p* = 0.002	*p* = 0.065
Recession (mm) Baseline	3.66	1.35	3.60	1.09			*p* = 0.832
Recession (mm) 6 mo Postoperatively	1.18	1.07	2.47	1.46			
6 mo—Baseline	−2.47	1.14	−1.13	0.94	*p* < 0.001	*p* < 0.001	*p* = 0.054
Keratinized Tissue Thickness (mm)	1.05	0.44	0.56	0.41			*p* = 0.044

## Data Availability

The data presented in this study are available on request from the corresponding author. The data are not publicly available due to privacy restrictions.
